# Effectiveness of accelerated perioperative care and rehabilitation intervention compared to current intervention after hip and knee arthroplasty. A before-after trial of 247 patients with a 3-month follow-up

**DOI:** 10.1186/1471-2474-9-59

**Published:** 2008-04-28

**Authors:** Kristian Larsen, Karen Elisabeth Hvass, Torben B Hansen, Per B Thomsen, Kjeld Søballe

**Affiliations:** 1 Orthopedic Research Unit, Regional Hospital Holstebro, Denmark; 2 University of Aarhus, Denmark; 3 Dept. of Orthopedics, Regional Hospital Holstebro, Denmark; 4 Dept. of Orthopedics, University Hospital of Aarhus, Denmark

## Abstract

**Background:**

In Denmark, approximately 12,000 hip and knee arthroplasties were performed in 2006, and the hospital costs were close to US$ 110,000,000. In a randomized clinical trial, we have recently demonstrated the efficacy of accelerated perioperative care and rehabilitation intervention after hip and knee arthroplasty compared to current intervention under ideal circumstances. We do not, however, know whether these results could be reached under usual circumstances of healthcare practice. We therefore investigated whether length of stay after implementation of accelerated perioperative care and rehabilitation after hip and knee arthroplasty could be reduced in a normal healthcare setting, and how the achieved results matched those observed during the randomized clinical trial.

**Methods:**

An effectiveness study as a before-after trial was undertaken in which all elective primary total hip and total knee arthroplasty patients were divided into a before-implementation group receiving the current perioperative procedure, and an after-implementation group receiving the new accelerated perioperative care and rehabilitation procedures as provided by a new multi-disciplinary organization. We used the Breakthrough Series Collaborative Model for implementation. The primary outcome measure was in hospital length of stay (LOS), and the secondary outcome measure was adverse effects within 3 months postoperatively.

**Results:**

We included a total of 247 patients. Mean LOS was significantly (*P *< 0.001) reduced by 4.4 (95% CI 3.8–5.0) days after implementation of the accelerated intervention, from 8.8 (SD 3.0) days before implementation to 4.3 (SD 1.8) days after implementation. No significant differences in adverse effects were observed. LOS in this effectiveness study was significantly lower than LOS reported in the efficacy study.

**Conclusion:**

Accelerated perioperative care and rehabilitation intervention after hip and knee arthroplasty was successfully and effectively implemented. Results obtained during usual hospital circumstances matched the results achieved under ideal circumstances in this group of patients.

## Background

Total hip and knee arthroplasties are the surgical treatments of choice when conservative treatments have failed for incurable pain in the hip and knee, the leading cause of which is osteoarthrosis [1]. In Denmark, the incidence of hip arthroplasty was estimated to be 142 per 100,000 inhabitants, and the incidence of knee arthroplasty was estimated to be 88 per 100,000 inhabitants in 2004 [2,3], and both incidences are rising [3,4]. In 2006, approximately 12,000 hip and knee arthroplasties were performed in Denmark [3]. In Denmark, the total hospital costs for hip and knee arthroplasties were close to US$ 110,000,000, based on the Danish diagnosis-related group (DRG) tariffs for 2005 [5].

New procedures to optimize perioperative procedures, in this study defined as procedures taking place in the period from hospitalization to discharge, have been given several different names, such as accelerated intervention, joint recovery program, multi-disciplinary intervention, multi-modal intervention, fast-track, and clinical pathway. The concept of an accelerated postoperative recovery program involves a coordinated effort to combine preoperative education of patients, preoperative optimization, attenuation of surgical stress response, optimized pain relief, enforced mobilization, nutritional support, and up-to-date postoperative nursing care and rehabilitation [6-9]. The concept of an accelerated postoperative recovery program has been developed to reduce perioperative and postoperative complications and to shorten the time needed for full recovery, especially after major surgical procedures [7]. In contrast, clinical pathways have been implemented in the United States of America (USA) in an effort to reduce length of hospital stay (LOS) and thereby control the hospital costs, whereas less focus has been placed on consequences for patients and society because of the introduction of new accelerated interventions [10]. Great differences are reported in LOS after total hip arthroplasty (THA), total knee arthroplasty (TKA), and unicompartmental knee arthroplasty (UKA) between hospitals in the USA and Europe [3,10]. Some of these differences could be explained by different motives for implementation.

The British pioneer clinical epidemiologist Archie Cochrane defined three concepts related to testing and implementing new healthcare interventions. 1) Efficacy is the extent to which an intervention does more good than harm under ideal circumstances ("Can it work?"). Ideally, the determination of efficacy is based on the results of a randomized clinical trial (RCT) [11]. 2) Effectiveness assesses whether an intervention does more good than harm when provided under usual circumstances of healthcare practice ("Does it work in practice?"). 3) Efficiency measures the effect of an intervention in relation to the resources it consumes ("Is it worth it?") [12]. Efficiency trials are more often called cost-effectiveness. We have followed this guideline, and have already demonstrated efficacy of accelerated perioperative care and rehabilitation intervention after hip and knee arthroplasty, and thereby answered the first of the three research question [13]. We still need to investigate whether the new accelerated intervention actually works under usual circumstances of healthcare practice.

Questions have been raised about the external validity of results obtained in RCTs of fast-track programs in this patient population because a large proportion of patients do not participate in the interventional studies [13]. Another problem when extrapolating results from efficacy studies to the target population is the Hawthorne effect (positive effect of being under study) [11], which potentially could affect both the healthcare staff and the patients. We therefore investigated whether it was possible to reduce LOS after implementation of accelerated perioperative care and rehabilitation intervention after hip and knee arthroplasty, and how results obtained in an effectiveness study corresponded to the results obtained in an efficacy study.

## Methods

### Study design

The study design was an effectiveness study performed as a prospective before-after trial. This clinical intervention trial followed the recommendations of the CONSORT Statement [14]. The study took place at the Orthopedic Clinic at Regional Hospital Holstebro in Denmark between January 2005 and April 2007. The procedures followed in the study were in accordance with the ethical standards of the responsible committee on human experimentation and with the Helsinki Declaration of 1975, as revised in 2000. The study protocol was approved by the Medical Ethics Committee of Ringkoebing and Southern Jutland Counties (Ref.:2627-04). The study was also registered in The Danish Data Protection Agency (J. no. 2004-41-4753), and the Clinical Trial Register (NCT00175201).

### Study subjects

All patients receiving a primary elective THA or TKA in the Regional Hospital Holstebro in the two study periods were consecutively included in the study. Patients receiving acute and revision surgery were excluded. Sample size was calculated from an alpha set at 0.05, a beta set at 0.95, average LOS estimated to be 8.0 days (SD 3.0) in the preimplementation period, and 6.5 days (SD 3.0) in the postimplementation period. At least 104 patients were therefore needed in both groups. For practical reasons, we decided that the two study periods would be of equal length, and we therefore included patients in the preimplementation period if they were operated on between January and April 2005 and in the postimplementation period if they were operated on between September and December 2006.

### Organization and interventions concerning both groups

#### Organization

In the preimplementation period the ward consisted of 36 beds, and the healthcare staff of 47 persons. This was reduced to 30 beds and a staff of 40 prior to our RCT [13], which was 1 year prior to the postimplementation period. The reduction in beds and staff did not affect patients receiving arthroplasty. Five physiotherapists and one occupational therapist managed rehabilitation. Approximately 20% of the ward, healthcare staff, and the rehabilitation staff were allocated to the arthroplasty patients. The number of working days in the ward was not changed during the study period. No unintentional change in staff-to-patient ratio occurred during the study period.

Six experienced surgeons performed all operations during the pre- and postimplementation period. The surgeons each did an equal number of arthroplasties (Additional file 1). Two surgeons, who did not perform THAs or TKAs were together with the six surgeons solely responsible for discharge. We took care that none of the organizational changes during the study period affected discharge procedures.

#### Intervention

Patients in both groups were subjected to identical operational procedures, defined as all procedures in the timeframe from leaving the ward for surgery till they were back in the ward after surgery. Operational procedures followed Danish guidelines [15,16]. No changes in surgical or anesthetic procedures from surgeons or anesthetists took place during the entire study period. Therefore the attenuation of the surgical stress response in both groups of patients was identical. There were furthermore no changes in post-discharge management.

Medication for pain relief was identical in the two groups. We used a visual analog scale (VAS) to measure pain. A VAS > 3 at rest and > 5 when active resulted in increased dose in pain-relieving medication. Preoperatively, we used paracetamol 1 mg. We used Oxycontin^® ^on the day of operation and the first day postoperatively. Doses were 10 mg 2 times daily for patients below 70 years of age, and 20 mg 2 times daily for patient at or above 70 years of age. VAS > 3 triggered supplementary opioids. From the second day postoperatively, we used Oxynorm^® ^5 mg if VAS > 3. We did not use patient-controlled analgesia (PCA) during the study period. We used Zofran^® ^4 mg for nausea reduction. Likewise there was no difference in use of continuous passive motion (CPM) during the entire study period, during which less than 2% of TKA patients used CPM.

The areas investigated in our accelerated intervention were therefore the changes in 1) the multi-disciplinary organization, and 2) the remaining elements from the multi-modal intervention [6-9]: preoperative assessment and information, optimization of oral nutrition from increased protein and fluid consumption, early and aggressive mobilization and exercise, hereafter defined as accelerated perioperative care and rehabilitation intervention.

### Organization and intervention in preimplementation period

Patients were hospitalized the day before surgery, and placed at random on the orthopedic ward. Patients were admitted the day before surgery for operations planned for Thursday to Friday, and on Friday if planned for Monday. Hospitalization took place on all days from Monday to Friday. A nutrition screening was performed on the day of admission, and patients were given food according to the result. The patients were given hospital garments to be worn during the whole stay, informed of the overall plan, and prepared for surgery. After surgery standard pain relief and nausea control procedures, as described above, were followed. The first day after surgery, the patients started training in bed before lunch, and were mobilized out of bed after lunch. After lunch, the patients were mobilized for the first time by a physiotherapist. During the stay, care was given in response to the patients' current needs. During the stay, mobilization was increased and adjusted according to the patients' immediate state in order to reach the discharge criteria. On average the patients were mobilized approximately 4 hours daily, starting with a few hours daily and increasing day by day until discharge. Mobilization consisted of all activities out of bed (50% of mobilization time), gait training (25% of mobilization time), and exercises (25% of mobilization time).

### Organization and intervention in postimplementation period

#### Multi-disciplinary organization

Patients were placed in separate male and female beds in the new nurse-lead multi-disciplinary accelerated care unit, which was placed in a separate part of the ward. Some intentional changes did occur in the organization. Surgery took place in the beginning of the week. Some nurse resources were moved from the end of the week to the beginning of the week and some hours on Mondays were moved from day to evening. All patients, accompanied by one relative, were invited to an information and preparation day the week before surgery. The purpose of the information day was not only to inform patients about the accelerated course of treatment, but also to prepare patients for surgery by individual consultation with surgeon, anesthetist, and nurse. Final blood tests, ECG, and radiographs were taken. Patients were hospitalized on the day of surgery.

#### Multi-modal intervention

In order to avoid patients adopting a sick role, they wore their own clothes during the whole stay. The healthcare staff worked to achieve written preset daily goals regarding: 1) information, 2) pain relief, 3) nausea control, 4) nutrition, 5) mobilization, and 6) elimination. 1) Information on the information day focused on goals during the hospital stay and a planned discharge on the fourth postoperative day if fulfillment of discharge criteria (sooner than fourth day if fulfilled or later if not), how to relieve pain, mobilization strategies, and providing them with delivering walking aid and other remedies. 2) Standard pain relief. 3) Standard nausea control. 4) A nutrition screening was performed on the information day, and patients were given food according to the result in addition to a daily intake of two protein beverages and a total fluid consumption of at least 1.5 liters. 5) Mobilization started on the day of surgery. The first postoperative day, the goal was 4 hours out of bed, including training with physiotherapist and occupational therapist. We tried to achieve more than 8 hours of mobilization per day for the rest of the hospital stay. Mobilization consisted of all activities out of bed (70% of mobilization time), gait training (15% of mobilization time), and exercises (15% of mobilization time). The physiotherapist was responsible for coaching the patient during exercises and gait training. Exercises focused on strengthening hip and knee muscles and how to avoid restricted movements. The exercises did not differ between the two intervention groups; however, there was much more focus on intensity, number of repetitions and progression in the accelerated intervention group. The patients were taught how to increase exercise and gait training after discharge. The occupational therapist was responsible for instruction regarding performance of personal needs for the THA patients. All healthcare staff members were aware of using all available situations for functional training, but also that the patients got needed time for rest. 6) For elimination, we used Magnesia^®^. Patients likewise followed a diary with the above-mentioned preset goals for nutrition, fluid consumption, and mobilization.

For further detailed information regarding the accelerated intervention, please see The Unit of Perioperative Nursing Care (homepage on the Internet) [17].

### Discharge in both groups

We used surgeons not otherwise involved in the study to decide in agreement with patients when discharge criteria were fulfilled. We tested the patients against the discharge criteria once daily in the morning, and only when the patient and the surgeons agreed on fulfillment of all criteria was the patient prepared for discharge.

The discharge criteria were the same in all patients groups during the whole study period except for the criteria of at least 90° of knee flexion in knee patients, which was not used in the postimplementation period. The discharge criteria were: 1) acceptance of discharge, 2) sufficient pain control, 3) aware of procedures for ending medication, 4) knowing the restrictions, 5) being able to correctly rise from lying and sitting 6) being able to walk safely with or without walking aids, 7) if necessary, being able to walk on stairs, 8) being able to perform home exercises, 9) knowing how to increase home exercises, 10) being able to perform personal needs, 11) helping aids delivered and installed, and 12) surgical wound showing no signs of infection 13) in knee patients, at least 90° of knee flexion (in the preimplementation period).

### Implementation method

We used the Breakthrough Series Collaborative Model, which consists of preparation, project and spread phases [18]. The spread phase to other wards and hospitals is currently ongoing, but is not reported in this study. We established an implementation organization, enrolled participants, and performed gradual implementation by using three learning sessions, three action periods, and three evaluation periods. Focus in all learning sessions and action periods was to develop an effective multi-disciplinary organization which in a proactive manner could master the multi-modal interventions [6-9]. After evaluation of the second activity period, which was performed as a RCT [19], the leading nurses who had developed the program handed over the new multi-disciplinary organization plan to new leading personnel, who were put in charge of the last full scale implementation in action period 3. Moreover, most of the healthcare staff involved in developing the new accelerated intervention was not part of the new postimplementation staff.

### Masking of healthcare staff and patients

The healthcare staffs in the pre- and postoperative periods were not aware of the ongoing study because all data were drawn from ongoing monitoring in the local and central hospital registers [3]. Likewise the patients were not aware of the ongoing study, because all contacts and questionnaires were part of the usual monitoring practice.

### Outcome measures

Primary outcome was in hospital LOS from admission to discharge. Secondary outcome measures were adverse effects (major perioperative complications, readmission within 30 days, and mortality within 3 months postoperatively). Data on all patients were collected via personal identification numbers, and postoperatively potential complications in these patients were sought in all Danish local and central hospital registers, which are available in closed databases.

### Statistics

The primary analysis was to test the difference in LOS between the current intervention observed in the preimplementation period and the fully implemented accelerated intervention in the postimplementation period. This analysis represents the effectiveness analysis of the accelerated intervention. Secondary analysis was to test the difference in LOS reported in the accelerated intervention group in the RCT by Larsen et al. [19] ("best case") with LOS obtained in the fully implemented procedures in the postimplementation period ("real case"), to see whether effectiveness could match efficacy. In addition, our goal was to test the difference between LOS obtained in the current intervention group in the preimplementation period (non-awareness of being under study) with LOS reported in the RCT by Larsen et al. [19] (awareness of ongoing study). LOS is presented with mean and standard deviation, together with the median and range. Because of the expectation of a non-normal distribution of LOS, the differences between groups were tested using Mann-Whitney rank sum test in the unadjusted analysis. In the multivariate analysis we used the non-parametric percentile method after a multivariate linear regression with 2000 non-parametric bootstrap replicates. The 95% confidence intervals were retrieved from 2000 bias-corrected and accelerated bootstrap replicates. A bootstrap simulation is a non-parametric method in which a random sample of the same size as the original sample is drawn several times with replacement from the original data. The differences in LOS were adjusted for gender, age, diagnosis, implant type, and patient group (THA, TKA). Categorical data were analyzed with Fisher's Exact test. The significance level was set at *P *< 0.05.

## Results

### Patient characteristics

**Figure 1:**
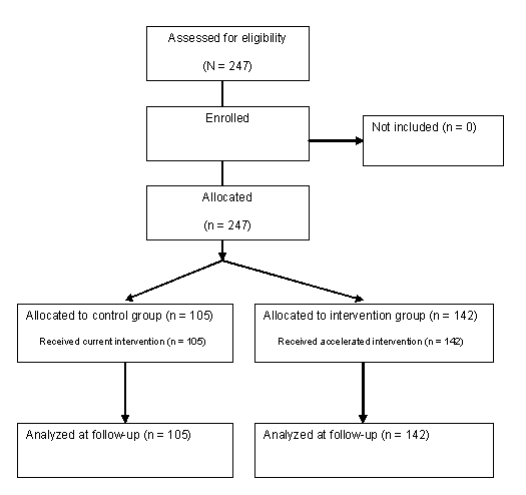
Flowchart of patients in effectiveness study.

A total of 105 patients were included in the preimplementation period, of which 15 were admitted on a Friday, and 142 patients were included in the postimplementation period. Complete data from all 247 patients receiving THAs and TKAs in the orthopedic clinic at the Regional Hospital Holstebro were available from admission to 3-month follow-up (Figure 1). Patient characteristic are presented in Additional file 1. No significant differences in patient characteristic between the two groups were observed. All patients met the discharge criteria before discharge.

### Length of stay

We observed a crude adjusted average reduction in LOS of 4.4 days (95% CI 3.8 – 5.0) from a LOS of 8.8 days (SD 3.0) for all patients receiving the current procedure in the preimplementation period to 4.3 days (SD 1.8) for all patients receiving the fully implemented accelerated intervention in the postimplementation period (*P *< 0.001). Mean LOS for the 15 patients admitted on a Friday was 9.8 days (SD 2.6), and mean LOS for the 90 patients admitted the day before surgery was 8.6 days (SD 3.0). The crude adjusted average reduction in LOS when excluding the 15 patients admitted on a Friday was 4.2 days (95% CI 3.7 – 4.9) (*P *< 0.001). Mean postoperative LOS (excluding LOS prior to the day of surgery) for the 105 patients in the preimplementation period was 7.5 days (SD 3.0). The crude adjusted average reduction in postoperative LOS was 3.1 days (95% CI 2.6 – 3.7) (*P *< 0.001). Crude LOS in the postimplementation period for the patients receiving the accelerated intervention was reduced to 4.0 days (SD 1.7) for the THA patients and 4.7 days (SD 1.7) for the TKA patients. For further information on unadjusted and adjusted crude and stratified results for LOS please refer to Additional file 2.

Crude LOS in the accelerated intervention group in the efficacy study by Larsen et al. was 5.0 (SD 2.4) (THA 4.3 (SD, 1.3), TKA 6.1 (SD, 3.5)) [19]. Compared to that result, we observed a significant further reduction in adjusted crude LOS of 0.8 (95% CI, 0.2 – 1.7), favoring the postimplementation period (*P *= 0.031).

In contrast, crude LOS in the current intervention group in the efficacy study by Larsen et al., in which the healthcare staff and patients were aware of the ongoing study, was 7.8 (SD 2.1) (THA 7.3 (SD, 1.5), TKA 9.3 (SD, 2.5)) [19]. Compared to that result, we observed a significantly longer adjusted LOS of 0.3 (95% CI 0.1–0.5) in the current intervention group in the preimplementation period, in which the healthcare staff and patient were not aware of the ongoing study (*P *= 0.015).

### Adverse effects

We registered only one major perioperative complication related to the implant in a THA patient in the postimplementation period. This complication, however, did not lead to a prolonged LOS.

No significant difference in number of patients readmitted within 30 days was observed. Five of 63 THA patients were readmitted in the preimplementation group, and 3 of 76 THA patients were readmitted in the postimplementation group (*P *= 0.472). Only 1 of 42 TKA patients was readmitted in the preimplementation group, versus of 3 out of 66 TKA patients in the postimplementation group (*P *= 1.0).

Likewise, no significant difference in mortality was observed, as only one patient in the preimplementation group, a 50-year old female THA patient, died perioperatively because of a respiratory arrest after pneumonia, and only one patient in the postimplementation group, a 85-year old female TKA patient, died 5 weeks after discharge, likewise after pneumonia (*P *= 1.0).

## Discussion

Our study revealed the successful implementation of accelerated perioperative care and rehabilitation intervention after hip and knee arthroplasty. We have further documented that LOS could be markedly reduced without increasing mortality and morbidity. Finally, we have documented that effectiveness could actually match efficacy in this patient population.

We believe that the observed reduction in adjusted LOS of 4.4 days between the preimplementation period and the postimplementation period was achieved by contribution from both parts of our accelerated intervention (i.e. changes in multi-disciplinary organization and multi-modal intervention). The change in admission procedure required the introduction of an information day, and was successfully implemented, although it was a great challenge and involved many departments. The new nurse-lead organization was the main factor responsible for the satisfactory function of the multi-disciplinary organization and acted in a more proactive manner than in previous systems because of clearly defined tasks and responsibility.

We believe the elements from the multi-modal intervention that contributed the most to the favorable results were the information day and the early and more aggressive mobilization, because there were no differences in operational procedures between the two intervention groups and only minor differences regarding pain relief, nausea reduction, nutrition and elimination.

When we compared the results from our efficacy study with the results from our effectiveness study, we expected that the average LOS in the accelerated intervention group in the efficacy would study to be shorter than the LOS in the postimplementation group in the effectiveness study because a best-case scenario is thought to be better than a real-case scenario. That the effectiveness result regarding LOS was actually significantly shorter than that in the efficacy study could have several explanations. One explanation is that our efficacy study was actually a pragmatic randomized clinical trial and a partial implementation under relatively normal circumstances, and not a "laboratory setup". This may have impaired the ultimately achievable result, which is therefore not known. We believe, however, that most of the difference was because the accelerated intervention was offered to all patients in the effectiveness study, whereas a rather high proportion of patients were not willing to participate in the efficacy study. These non-participating patients consisted of at least two different groups, one of which was a group of younger patients receiving uncemented arthroplasties, which we have shown to have the shortest LOS [19]. The differences we observed between the results from the efficacy and the effectiveness study could indicate that there are problems in extrapolating results from RCTs with high proportions of refusing patients to the target population. This observation is in accordance with Petersen et al., who along with others, have questioned the results from RCTs with rather large proportions of non-participating patients [13]. The difference in results between the two study designs could not be explained by a mere extension of the accelerated intervention from the efficacy to effectiveness study, because all leading members of healthcare staff and most other members of the other healthcare staff differed between the two study periods.

When we compared the results obtained in the control groups in the efficacy study and the effectiveness study, there was some indication of a possible but small Hawthorne effect in the efficacy study, because we had ruled out contamination in the control group in the efficacy study by a structured observation [19]. The result for LOS in the control group in the efficacy study, in which patients and healthcare staff were aware of being under study, thereby potentially increasing attention to objectives of the study, was significantly lower than that observed in the effectiveness study, in which they were not aware of being under study [19]. This observation is in line with the results presented in the RCT by Dowsey et al., in which they reported a large reduction in LOS in both the intervention and control groups compared to the period just prior to the study period [20]. The Hawthorne effect could potentially have been even larger because we would have expected a small increase in LOS in the control group in the RCT because of a higher proportion of patients admitted on Fridays, because surgery in this period was done at the beginning of the week.

The observed reduction in LOS from preimplementation period to postimplementation period in our study is in accordance with three of the four published efficacy studies [19-21], but in conflict with the study by Petersen et. al [22]. The result is also in accordance with other Danish effectiveness studies [23-27]. It also corresponds well with the results reported in the review by Kim et al. [28] and in the Dutch cost-effectiveness study by Brunenberg et al. [29]

The observed number of complications is also in accordance with a comparable publication, in which a tendency towards fewer complications for hip patients and more complications for knee patients is reported [30].

Have we reached the limit with our implementation regarding LOS after accelerated procedures for hip and knee arthroplasty? Apparently not, because the study by Walter et al. indicates that it could be possible to further accelerate the convalescence of these patient groups because they reported an average LOS of 3.2 days for THA patients and 3.0 for TKA patients by using a newly designed clinical pathway [31]. But on moving from accelerated to super accelerated procedures, we have to be extremely cautious because of serious early postoperative complications [32]. Because an intervention aiming at discharge within 3 days is not the same as an intervention aiming at discharge within 7 days, we propose the following definitions for future use in patients receiving primary elective THA, TKA, and UKA: Super-accelerated intervention is defined as an intervention with planned discharge within 3 days. Accelerated intervention is defined as an intervention with planned discharge within 5 days. Semi-accelerated intervention is defined as an intervention with planned discharge within 7 days. Non-accelerated intervention is defined as an intervention with an average LOS of more than 7 days. These definitions are in line with the definition used in the Danish Health Technology Assessment for THA and TKA [30].

We consider the quality of our data to be good, since all data used were obtained from available official Danish databases, which have been in use for several years and been through some form of validation process [33]. We also searched data from all Danish hospitals in order not to miss patients readmitted to hospitals outside our own region.

We observed a reduction in beds and healthcare staff from preimplementation period to postimplementation period, which potentially could have affected the results through pressure on change in discharge procedures. However, the reduction in beds and staff did not concern arthroplasty patients, and we do not believe that it had any effect on discharge because the concerned healthcare staff was not involved in discharge, which was performed by surgeons not otherwise involved in the study.

Identical discharge criteria were a core principle in this study because LOS was both related to intervention and outcome. We did, however, omit the criterion of at least 90° of knee flexion in knee patients in the postimplementation period. This was done because we found that this criterion was an unnecessary prerequisite for patients to be mobilized and to function well in the RCT by Larsen et al. [19]. Omission of this criterion is in accordance with the Danish Health Technology Assessment for THA and TKA [30]. We believe the omission of this criterion has led to a reduction in LOS of approximately 1.5 days, because LOS for TKA in our RCT [19], in which the intervention was identical but the criterion was included had a LOS of 6.1 days, whereas LOS was 4.7 days in the postimplementation period, in which this criterion was omitted.

When we calculated LOS we used in hospital LOS from admission to discharge, which is the actual time a patient occupies a bed in the ward. The observed difference in LOS therefore consisted of two elements, which we considered to be equally important parts of our accelerated intervention, namely a reduction in LOS due to changed admission procedures in the multi-disciplinary organization, and a reduction in LOS due to changed multi-modal intervention. It was not the purpose of this study to distinguish between these two elements. But in our RCT, we observed a reduction in LOS because of a change in the organization of admission procedures to account for a mean reduction of 1.5 days [19], and we have shown that this result also applies to this study.

When we estimated the reduction in LOS between groups, we used multivariate analysis in order to adjust for potential differences in the most important covariates to get the most precise estimate. In our multivariate analysis the differences in LOS were adjusted for gender, age, diagnosis, implant type, and patient group (THA, TKA), which are considered important patient characteristics to describe case mix [34,35]. Other relevant patient characteristics are American Society of Anaesthesiology Score (ASA) and blood transfusion [36]. However, we were not able to include ASA scores because of invalid and incomplete data, and did not include blood transfusion because the operational procedure was not part of the intervention.

Costs and consequences for other healthcare sectors owing to changes in interventions in the hospital are indeed relevant, and these questions will be answered in ongoing cost-efficacy and cost-effectiveness studies.

It is, however, a limitation of the current study that there were no patient-reported outcome data or functional performance tests. We intended to include the patient-specific outcome measures, which are reported to the Danish Hip and Knee Arthroplasty registers, but we found these data to be invalid and incomplete. We do, however, know from our own RCT, that TKA patients reports equal health-related quality-of-life (HRQOL) 3 months postoperatively, irrespective of receiving standard or accelerated interventions, while THA patients receiving accelerated intervention report a HRQOL 3 months postoperatively that is approximately 10% higher that patients receiving the non-accelerated intervention [19]. We are currently performing a study in which we investigate whether this observation regarding HRQOL observed in our RCT also applies when we compare the preimplementation period with the postimplementation period.

In summary, we still need to evaluate results from efficacy, effectiveness, and efficiency studies before accepting a new program of intervention. Special care must though be taken in the future to minimize the proportion of non-participating patients in efficacy studies, because we could miss the ultimately achievable results. We must include information on as many potential confounders as possible in effectiveness studies in order to reduce bias, and we must also investigate the costs and consequences outside the hospital due to changes in hospital interventions.

## Conclusion

Accelerated perioperative care and rehabilitation intervention after hip and knee arthroplasty was successfully and effectively implemented. Results obtained during usual hospital circumstances matched the results achieved under ideal circumstances in this group of patients.

## Competing interests

The authors declare that they have no competing interests.

## Authors' contributions

KL, KEH and TBH conceived of the study, and participated in its design and coordination and helped to draft the manuscript. KL performed the statistical analysis. All authors contributed to the planning, interpretation and revision of the manuscript. All authors read and approved the final manuscript.

## Pre-publication history

The pre-publication history for this paper can be accessed here:



## Supplementary Material

Additional file 1 **Table 1.** Patient characteristic at baseline for 247 patients in the current and accelerated intervention groups. Click here for file

Additional file 2 **Table 2.** Unadjusted and adjusted crude and stratified difference in length of stay for 247 patients in the two intervention groups receiving THA* and TKA† Click here for file
