# Supplementary material for: Effectiveness of accelerated perioperative care and rehabilitation intervention compared to current intervention after hip and knee arthroplasty. A before-after trial of 247 patients with a 3-month follow-up
Source: BMC Musculoskelet Disord. 2008 Apr 28;9:59. doi: 10.1186/1471-2474-9-59 (PMC2396162; doi:10.1186/1471-2474-9-59)
Supplement: Additional file 1 — Table 1. Patient characteristic at baseline for 247 patients in the current and accelerated intervention groups. [file 1471-2474-9-59-S1.doc]

Table 1. Patient characteristic at baseline for 247 patients in the current and accelerated intervention groups

Current Accelerated *P* value

intervention intervention

( n = 105) ( n = 142)

Gender, female vs. male 52 / 53 74 / 68 0.687

Age, mean and standard deviation 65 (11.0) 65 (11.0) 0.837

Diagnosis, arthrosis vs. other 97 / 7 139 / 3 0.073

Implant type, cemented vs. uncemented 37 / 68 41 / 101 0.287

Patient group (ratio THA : TKA)* 63 : 42 76 : 66 0.310

Surgeon A (ratio THA : TKA) 13 : 8 19 : 10 0.793

Surgeon B (ratio THA : TKA) 14 : 7 17 : 11 0.669

Surgeon C (ratio THA : TKA) 14 : 8 19 : 9 0.754

Surgeon D (ratio THA : TKA) 2 : 19 3 : 26 0.924

Surgeon E (ratio THA : TKA) 20 : 0

Surgeon F (ratio THA : TKA) 18 : 10

^*^ Total hip arthroplasty, total knee arthroplasty
