# Supplementary material for: Effectiveness of accelerated perioperative care and rehabilitation intervention compared to current intervention after hip and knee arthroplasty. A before-after trial of 247 patients with a 3-month follow-up
Source: BMC Musculoskelet Disord. 2008 Apr 28;9:59. doi: 10.1186/1471-2474-9-59 (PMC2396162; doi:10.1186/1471-2474-9-59)
Supplement: Additional file 2 — Table 2. Unadjusted and adjusted crude and stratified difference in length of stay for 247 patients in the two intervention groups receiving THA* and TKA† [file 1471-2474-9-59-S2.doc]

Table 2. Unadjusted and adjusted crude and stratified difference in length of stay for 247 patients in the two intervention groups receiving THA* and TKA^†^

Current Accelerated Difference *P* value^§^

intervention intervention mean, CI^‡^

Unadjusted

Crude (n) 105 142

Mean (SD) 8.8 (3.0) 4.3 (1.8) 4.4 (3.8 – 5.0) < 0.001

Median (Range) 8 (4-21) 4 (2-11)

Stratified

THA, (n) 63 76

Mean (SD) 8.4 (3.3) 4.0 (1.7) 4.4 (3.5 – 5.3) < 0.001

Median 7 (4-21) 4 (2-11)

TKA, (n) 42 66

Mean (SD) 9.4 (2.4) 4.7 (1.7) 4.6 (3.8 – 5.4) < 0.001

Median 8.5 (6-15) 4 (2-11)

Adjusted difference

Crude (N = 247) 4.4 (3.8 – 5.0) < 0.001

Stratified

THA (n = 139) 4.3 (3.6 – 5.2) < 0.001

TKA (n = 108) 4.6 (3.8 – 5.5) < 0.001

* Total hip arthroplasty

^†^ Total knee arthroplasty

^‡^ 95% confidence intervals

^§^ Difference between groups tested with Mann-Whitney non-parametric test in the unadjusted analysis and with non-parametric percentile method in the adjusted analysis
